# Effects of Exercise Training on Fat Loss and Lean Mass Gain in Mexican-American and Korean Premenopausal Women

**DOI:** 10.1155/2017/5465869

**Published:** 2017-07-06

**Authors:** Shenghui Wu, Kyung-Shin Park, Joseph B. McCormick

**Affiliations:** ^1^Department of Epidemiology & Biostatistics, University of Texas Health Science Center at San Antonio-Laredo Campus, Laredo, TX 78041, USA; ^2^Kinesiology, Texas A&M International University, 5201 University Blvd., Laredo, TX 78041, USA; ^3^Division of Epidemiology, School of Public Health, Brownsville Campus, University of Texas Health Science Center-Houston, Brownsville, TX 78520, USA

## Abstract

We investigated the effect of exercise training on body composition change in women. Nineteen Mexican-American and 18 Korean premenopausal overweight/obese women were randomized into one of the following groups: control, low-intensity training group (LI), and high-intensity training group (HI). Subjects completed 12 weeks of training at 50–56% maximal oxygen consumption (LI) or 65–70% maximal oxygen consumption (HI). Body composition components were measured at baseline and after training using dual-energy X-ray absorptiometry for Mexican-Americans, while whole-body composition was measured by the direct segmental multifrequency bioelectrical impedance analysis and abdominal fat was measured by single-slice computed tomography for Koreans. Data were analyzed using mixed-model repeated measures independent of age, ethnicity, and body mass index (BMI). Exercise training showed a significant effect on BMI, fat percentage, fat mass, lean mass, and visceral adipose tissue area. HI significantly decreased fat mass and fat percentage but increased lean mass (all *P* < 0.05). LI significantly reduced BMI, fat mass, fat percentage, and visceral adipose tissue area but increased lean mass (all *P* < 0.05). Exercise training had a beneficial effect on reducing BMI, fat percentage, fat mass, and visceral adipose tissue area but had no effect on increasing lean mass for Mexican-American and Korean premenopausal overweight/obese women.

## 1. Introduction

Overweight/obesity increases the risk for many diseases such as cancers, cardiovascular disease, and type 2 diabetes mellitus [1]. Previous studies have shown that exercise intervention such as moderate endurance training decreases body mass index (BMI), total fat mass, or percent fat [2, 3], but only a limited number of studies have reported the effect of exercise training on body composition in minorities. A randomized clinical trial reported that yoga exercise significantly decreased body weight, percentage of body fat, BMI, waist circumference, and visceral fat area measured by using the bioelectrical impedance analysis method in 16 postmenopausal obese Korean women in a clinical trial, but lean body mass did not change [4]. Walking for 30 min 3 days a week resulted in reductions in body fat estimated by skinfold sums among premenopausal overweight Mexican-American women [5]. Minority women have relatively higher risks of morbidity and mortality because of obesity and sedentary activity; therefore, they would benefit the most from exercise intervention in the reduction of these health risks and would be more likely to participate in exercise [6]. The purpose of this study was to investigate the effect of exercise training under relatively equal energy expenditure on body composition change in Korean and Mexican-American premenopausal women.

## 2. Materials and Methods

### 2.1. Subjects

Subjects were apparently healthy overweight or obese females between the ages of 30 and 50 years. Participants were considered eligible if they were nonsmokers, premenopausal, overweight, or obese (BMI ≥ 30 kg/m^2^); did not have any known chronic disease; did not take medication that would alter metabolic, cardiovascular, or immune function; and had no musculoskeletal limitations. Participants who did not have menses during the last 2 months were excluded from this study. A total of 19 Mexican-American recruited from the city of Laredo, Texas, US, and 18 Korean subjects recruited from the metropolitan area, Korea. This study was approved by the Institutional Review Board from the Texas A&M International University (TAMIU IRB 20100121) and the University of Texas Health Science Center. Participants provided written informed consent.

### 2.2. Study Design

Nineteen Mexican-American and eighteen Korean eligible women were randomized into one of the following groups: (1) no exercise training (control), (2) low-intensity exercise training (LI, 50–56% VO_2_max), and (3) high-intensity exercise training (HI, 65–70% VO_2_max). Subjects were assessed before and after the 12-week intervention. Subjects were asked to maintain their diet and lifestyle habits during the intervention period.

All body composition indicators were measured and assessed before and after the 12 weeks of intervention. For each test, subjects arrived at the testing center between 0800 and 0900 h after 12 h of fasting and underwent anthropometric measurements, body fat and central fat assessments, and VO_2_max test. Measurements and assessments were undertaken at the same phase of the menstrual cycle for each subject before and after the intervention.

### 2.3. Anthropometric Measurements

All measurements were completed in duplicate by a trained technician. Height and weight were measured to the nearest 0.1 cm and 0.1 kg, respectively, with the participants wearing indoor clothes without shoes.

### 2.4. Body Composition Assessment

Total and multicompartment body composition was measured by dual-energy X-ray absorptiometry (DXA) (Hologic Discovery Series, Bedford, Massachusetts) for Mexican-American subjects. This allows for the estimation of total lean mass and fat mass within specific regions, including the visceral depot. Subjects lay on the table and the entire body was scanned. The subject lay flat on her back for the duration of the scan without difficulty, pain, or shortness of breath. Subjects were asked to wear a gown and to remove all metal objects (glasses, jewelry, and cell phones). Subjects were exposed to a small dose of radiation (20 DXA = 1 chest X-ray, average dose from DXA scan: 0.1 mrem to each participant per time). The scans were performed following the standard clinical protocol.

For Korean subjects, whole-body composition was measured using direct segmental multifrequency bioelectrical impedance analysis (Inbody 3.0, Biospace, Seoul, South Korea). The measurements were performed after 12 h of fasting and within 30 min of voiding the urinary bladder. Subjects were also instructed to drink a bottle of water (500 mL) an hour before sleeping and 2 h before the body composition measurements to maintain well-hydrated state. For abdominal fat, single-slice computed tomography (CT: ECLOS, Hitachi, Tokyo, Japan) images were obtained from L4-L5 intervertebral disc space to measure visceral abdominal fat area (VFA). The mean value of all pixels within the range of −150 to −50 Hounsfield units was determined using a commercial software program (Fat pointer, Hitachi). CT image acquisition and the measurement of abdominal fat area were performed by different technicians who were working in the university medical center in a blinded fashion.

### 2.5. VO_2_max Test

VO_2_max was measured once before training to set exercise intensity and duration of 12 weeks of training for each subject. VO_2_max was measured during a continuous, progressive, treadmill-running protocol (modified Bruce Protocol). VO_2_max was assumed if subjects attained at least 2 of the following criteria: (i) volitional fatigue, (ii) an increase in workload with little or no increase in heart rate and oxygen consumption, and (iii) respiratory exchange ratio greater than 1.1. A trained technician asked the Borg rating of perceived exertion to the subject in each stage to check volitional fatigue and monitored changes of oxygen consumption and respiratory exchange ratio for the second and third criteria.

### 2.6. Exercise Intervention

Subjects in LI and HI performed 12 weeks of training at the given exercise intensity. We used the heart rate each subject obtained at the given exercise intensity (50% and 70% VO_2_max, resp.) during VO_2_max test; therefore, subjects were asked to walk or run at the treadmill speed where they could maintain this target heart rate during exercise. Each subject performed five exercise sessions per week. The duration of each exercise session was adjusted on the basis of each subject's VO_2_-velocity relationship; each subject expended 13.5 METs·h/week (14.2 kcal·kg^−1^·week^−1^), 18 METs·h/week (18.9 kcal·kg^−1^·week^−1^) for weeks 4–8 (4 sessions per week), and 22.5 METs·h/week (23.6 kcal·kg^−1^·week^−1^) for the last 4 weeks (5 sessions per week). As each subject's fitness level improved, the running speed on treadmill was increased on the basis of heart rate. In each training session, heart rate was measured telemetrically every 5 min (Polar, Oy, Finland) and average heart rate was calculated. Running speed was adjusted when average heart rate was decreased by more than 5 beats per minute on 2 consecutive training sessions. Weekly target energy expenditure (EE) for each subject was calculated using the following equation [7]:

(1) $$\begin{array}{c} EE=\left(predetermined\;METs\cdot h/week\times \left(BW\times 60\times 3.5\times \frac{5}{1000}\right)\right). \end{array}$$

The duration of each exercise session was adjusted on the basis of each participant's VO_2_max using the following equation [7] to equate energy expenditure relative to kilograms of body weight between HI and LI groups:

(2) $$\begin{array}{c} Duration=\frac{EE}{\left[\left(V\times I\right)/1000\times 5\times F\times BW\right]}, \end{array}$$

where predetermined METs·h/week were 13.5, 18, and 22.5, as described above. In the same equation, 60 is the number of minutes in an hour, 3.5 (mL·kg^−1^·min^−1^) is the resting oxygen consumption, 5/1000 (kcal/L O_2_) is the energy expenditure for oxygen consumption per mL, *V* is the VO_2_max (mL·kg^−1^·min^−1^), *I* is the exercise intensity expressed as a fraction, *F* is the exercise frequency (times·week^−1^), and BW is the body weight in kg.

### 2.7. Statistical Analysis

Descriptive analyses were conducted to compare the characteristics of the subjects among control, low-intensity exercise, and high-intensity exercise groups. Log-transformed data were conducted to normalize the distribution of continuous variables as appropriate. Mixed-model repeated measures were used to detect the difference in body composition indices at baseline and after exercise training among the 3 groups. The models were controlled for age, ethnicity, and BMI. Statistical analyses were carried out by using SAS version 9.4 (SAS Institute, Cary, NC). All statistical tests were based on 2-sided probability. Statistical significance was accepted for all tests at *P* < 0.05.

## 3. Result

The characteristics of 37 subjects at baseline and after 12 weeks' interventions are presented in Table 1. All except 3 subjects (2 in HI and 1 in LI) completed 48 sessions of training in 12 weeks; however, these three subjects also completed 48 sessions of training either at the 13th week (1 in HI and 1 in LI) or at the 14th week (1 in HI). Therefore, all of them were included in data analyses. All these exercise sessions were supervised by the trainers. We asked subjects to work out at their convenience from morning to night through seven days per week by the appointment. If subjects missed their appointments, we made new appointments to complete the given exercise session.

All of the 19 Mexican-American and 18 Korean women were not cigarette smokers or alcohol drinkers. The average age is 28.4 (standard deviation: 6.4) years. The groups did not differ at baseline except for BMI for which LI has the lowest value. Exercise training showed statistically significant effect on weight (*P* = 0.0004), BMI (*P* = 0.0004), fat percentage (*P* < 0.0001), fat mass (*P* < 0.0001), visceral adipose tissue area (*P* = 0.02), and total mass (*P* = 0.001). High-intensity training for 12 weeks significantly decreased body weight (*P* = 0.0005), BMI (*P* < 0.0001), fat mass (*P* = 0.002), total mass (*P* = 0.0008), VAT mass for Mexican-Americans (*P* = 0.03), and VAA for Koreans (*P* = 0.013) but increased lean mass (*P* < 0.0001). HI significantly reduced weight, BMI, and total mass but increased lean mass (all *P* < 0.05). There were no other significant changes in control. The average running time per session and running speed obtained in the last week were 35.4 ± 2.3 min and 7.0 ± 0.6 km·h^−1^, respectively, for HI and 47.3 ± 4.9 min and 5.1 ± 0.6 km·h^−1^, respectively, for LI in Mexican-American subjects and 34.5 ± 1.5 min and 7.42 ± 0.45 km·h^−1^, respectively, for HI and 41.8 ± 1.95 min and 5.92 ± 0.34 km·h^−1^, respectively, for LI in Korean subjects.

**Figure 1:**
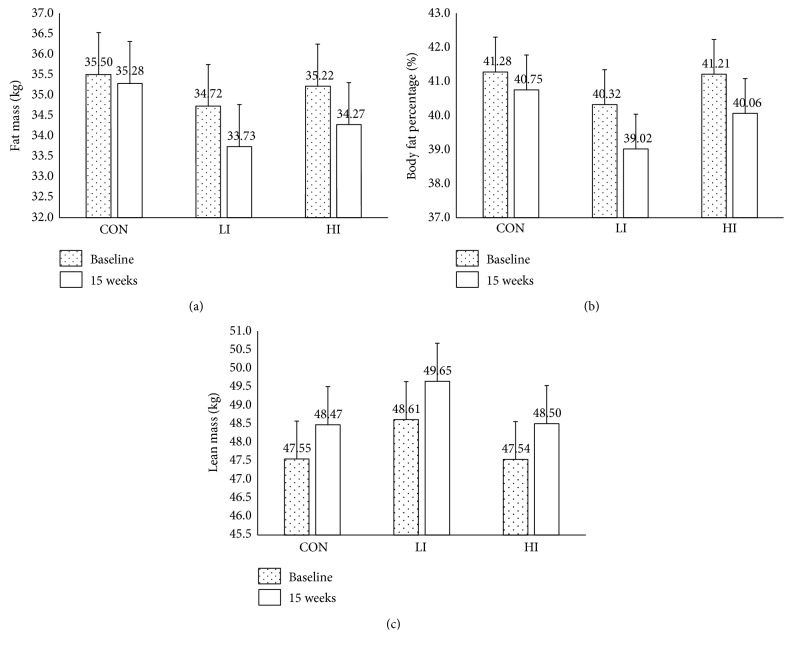
Multivariable-adjusted means and standard errors of fat mass (a), body fat percentage (b), and lean mass (c) at baseline and after 12 weeks of low- or high-intensity exercise training, that is, control (CON), low-intensity (LI), and high-intensity training (HI). There were no significant differences between the groups at baseline, but the groups were significantly different between baseline and 12 weeks in each group (all *P* < 0.05).

After adjusting for age, BMI, ethnicity, and interaction terms, HI significantly decreased fat mass (*P* = 0.0002) and fat percentage (*P* < 0.0001) but increased lean mass (*P* = 0.0007). LI significantly reduced BMI, fat mass, fat percentage, and visceral adipose tissue area but increased lean mass (all *P* < 0.05) (Table 2). The changes in fat mass, body fat percentage, and lean mass following 12 weeks of high- or low-intensity exercise training are shown in Table 2 and Figure 1. Fat mass and body fat percentage increased significantly after 12 weeks of low-intensity or high-intensity exercise training. A significant increase in lean mass was also observed in the LI and HI groups. No statistically significant difference of changes in the same indicator was found between the LI and HI groups. No other significant changes were observed in the control group.

## 4. Discussion

Our study investigated the effect of exercise training on body composition change in women. Exercise training showed statistically significant effect on BMI, fat percentage, fat mass, lean mass, and visceral adipose tissue area. Independent of age, BMI, and ethnicity, HI significantly decreased fat mass and fat percentage but increased lean mass, and LI significantly reduced BMI, fat mass, fat percentage, and visceral adipose tissue area but increased lean mass. These data give strong indications that exercise training had a beneficial effect on improving body composition.

To our knowledge, our study is the first to investigate the effect of exercise training under relatively equal energy expenditure on body composition change in Korean and Mexican-American premenopausal women. Only several previous studies on the effect of exercise training were conducted among Mexican-Americans or Koreans. A trial with 22 Korean premenopausal overweight/obese women showed that 14 weeks of high-intensity exercise training is more beneficial in whole body and abdominal fat loss [8]. However, that study used bioelectrical impedance analysis to measure body composition, while our study used DXA measurement. The accuracy of the bioelectrical impedance machine has been questioned [8], while DXA has good validity and excellent precision [9]. Another two studies with moderate intensity of exercise training showed that yoga exercise significantly decreased body weight, percentage of body fat, lean body mass, BMI, waist circumference, and visceral fat in 16 postmenopausal obese Korean women [4] and walking for 30 min 3 days a week resulted in reductions in body fat among premenopausal overweight Mexican-American women [5]. However, our study showed that LI seems to be more effective in body weight and visceral adipose than HI. Also, Coker et al. found that 12 weeks of high-intensity training (75% VO_2_max) resulted in a significant reduction in visceral fat, but not in subcutaneous abdominal fat [10]. Clearly, the further research on the different effects on body composition between LI and HI should be investigated.

Several physiological explanations might be related to the effect of exercise training on the reduction of obesity. In obesity, circulating levels of inflammatory markers are elevated [11]. Exercise training reduces chronic inflammation through its effect on muscle tissue to generate muscle-derived, anti-inflammatory “myokine,” its effect on adipose tissue to improve hypoxia and reduce local adipose tissue inflammation, its effect on endothelial cells to reduce leukocyte adhesion and cytokine production systemically, and its effect on the immune system to lower the number of proinflammatory cells and reduce proinflammatory cytokine production per cell [11]. It is very likely that exercise training stimulates adipose tissue angiogenesis and increases blood flow, thereby reducing hypoxia and the associated chronic inflammation in adipose tissue of obese individuals [11]. Physical activity probably does not lead to an altered myokine response, which could provide a potential mechanism for the association between sedentary behavior and many chronic diseases including obesity [12]. Resistance exercise may provide a therapeutic target for releasing endogenous growth hormone in individuals who are obese, and biological activity of growth hormone indicates that this may be an important precursor to beneficial changes in body fat and lean tissue mass in obese individuals [13]. Physical activity may represent a complementary alternative approach for the clinical management of endocannabinoid system deregulation in obesity, without the side effects occurring with CB1 receptor antagonists [13].

This study had several strengths. First, it was a randomized controlled trial involving 12 weeks of exercise training. Second, weekly energy expenditure was equated relative to body weight using METs·h/week, which allowed subjects to conduct relatively equal amounts of exercise regardless of their body weight. Finally, we used the best available methods to measure body composition. The direct segmental multifrequency bioelectrical impedance method was used to measure whole-body composition for Korean subjects. Recent studies reported that there is a small differences (less than 4%) between the multifrequency bioelectrical impedance method and DXA [14], with the percentage coefficient of reliability for the multifrequency bioelectrical impedance higher than 97% [15], indicating the utility of this method in monitoring longitudinal changes in body composition. To estimate visceral adipose tissue area/mass, we used single-slice computed tomography for Korean and DXA for Mexican-American subjects. Single-slice computed tomography is one of the current gold standards for quantifying visceral fat, and current investigations show strong agreement between DXA and CT in visceral adipose fat measurement with the coefficient of determination (*r*^2^) for regression of CT on DXA values of 0.959 for females [16].

There are some limitations in our study. First, daily diet intake and physical activities were not controlled in this study. We asked subjects to maintain their lifestyle during 12 weeks of training intervention, but we did not control or observe their daily diet intake and energy expenditure. Second, we did not collect other covariates such as socioeconomic status due to limited time, cost, and personnel. Third, we had to use different equipment in the measurement of whole-body composition and visceral adipose tissue from Mexican-American and Korean female subjects due to the absence of equipment in two different testing centers. Another limitation in this study was that %VO_2_max is not a common method for prescribing intensity and fails to account for differences in fitness levels. Thus, the method for exercise intensity might produce relatively inaccurate results.

## 5. Conclusions

Exercise training had a beneficial effect on reducing BMI, fat percentage, fat mass, and visceral adipose tissue area but had no effect on increasing lean mass for Mexican-American and Korean premenopausal obese women. Further research on the effect of intensity of exercise training is still warranted.

## Figures and Tables

**Table 1:** Characteristics and body composition at baseline and after 12 weeks of low- or high-intensity exercise training.

	Control (*n* = 12)			Low-intensity exercise (*n* = 11)			High-intensity exercise (*n* = 14)			*P* for effect of group	*P* for effect of exercise
	Baseline	After 12 weeks	*P*	Baseline	After 12 weeks	*P*	Baseline	After 12 weeks	*P*		
Continuous variables [mean (SD)]											
Age, years	28.83 (5.51)	28.92 (5.66)	0.60	25.82 (5.27)	26.09 (5.39)	0.10	30.07 (7.69)	30.36 (7.84)	0.06	—	—
Height, cm	161.68 (3.13)	—	—	161.42 (4.86)	—	—	160.11 (5.69)	—	—	—	—
Weight, kg	95.48^∗^ (17.43)	95.37^∗^ (17.5)	0.01	83.07^∗^ (6.1)	79.97^∗^ (6.44)	0.0005	89.8 (14.4)	87.69 (14.66)	0.01	0.06	0.0004
BMI, kg/m^2^	36.49 (1.37)	36.44^∗^ (1.39)	0.56	31.83 (1.43)	30.65^∗^ (1.45)	<0.0001	34.97^∗^ (1.27)	34.11^∗^ (1.28)	<0.0001	0.03	0.0004
Body fat, %	42.61 (6.74)	41.97 (6.02)	0.053	39.37 (4.73)	37.69 (4.86)	0.21	42.3 (4.33)	40.83 (4.38)	0.82	0.21	<0.0001
Fat mass, kg	40.26 (12.88)	39.77^∗^ (12.03)	0.88	32.09 (4.97)	29.61^∗^ (4.93)	0.002	37.42 (8.56)	35.25 (8.58)	0.008	0.06	<0.0001
Lean mass, kg	50.30 (5.77)	51.31 (6.82)	0.07	47.23 (5)	46.71 (5.09)	<0.0001	48.17 (6.96)	48.07 (6.95)	<0.0001	0.35	0.07
Visceral adipose tissue mass for Mexican-Americans, kg	0.90 (0.23)	0.82^∗^ (0.23)	0.18	0.64 (0.18)	0.58^∗^ (0.03)	0.37	0.84 (0.23)	0.78 (0.23)	0.22	0.12	0.06
Visceral adipose tissue area for Koreans, cm^2^	80.9 (14.5)	84.7 (13.2)	0.47	71.4 (18.2)	53.0 (16.3)	0.003	81.7 (32.3)	72.1 (18.0)	0.08	0.18	0.02
Total mass, kg	93.32 (17.09)	92.82^∗^ (16.63)	0.66	78.45 (6.31)	81.39 (6.18)	0.0008	85.69 (14.27)	87.95 (14.13)	0.006	0.07	0.001
Categorical variables, number (%)											
Mexican-Americans	6 (50%)	6 (50%)	—	5 (45.45%)	5 (45.45%)	—	8 (57.14%)	8 (57.14%)	—	—	—
Koreans	6 (50%)	6 (50%)	—	6 (54.55%)	6 (54.55%)	—	6 (42.86%)	6 (42.86%)	—	—	—

^∗^Significantly different from the control group at the same time period (either at baseline or after 12 weeks for both comparison groups).

**Table 2:** Multivariable-adjusted body composition at baseline and after 12 weeks of low- or high-intensity exercise training.

Measures (mean [SE])	Control (*n* = 12)			Low-intensity exercise (*n* = 11)			High-intensity exercise (*n* = 14)			*P* for effect of group	*P* for effect of exercise
	Baseline	After 12 weeks	*P*	Baseline	After 12 weeks	*P*	Baseline	After 12 weeks	*P*		
Weight, kg	88.49 (1.02)	88.46 (1.02)	0.38	88.23 (1.02)	88.19 (1.02)	0.19	86.79 (1.02)	86.83 (1.02)	0.25	0.68	0.53
BMI, kg/m^2^	36.05 (1.03)	35.98 (1.03)	1.00	32.17 (1.04)	30.95 (1.04)	0.03	34.37 (1.03)	33.5 (1.03)	0.12	0.03	0.0004
Body fat, %	41.28 (1.02)	40.75 (1.02)	0.04	40.32 (1.02)	39.02 (1.02)	<0.0001	41.21 (1.02)	40.06 (1.02)	<0.0001	0.57	<0.0001
Fat mass, kg	35.5 (1.03)	35.28 (1.03)	0.36	34.72 (1.03)	33.73 (1.03)	0.0008	35.22 (1.03)	34.27 (1.03)	0.0002	0.73	<0.0001
Lean mass, kg	47.55 (1.02)	48.47 (1.02)	0.001	48.61 (1.02)	49.65 (1.02)	0.002	47.54 (1.02)	48.50 (1.02)	0.0007	0.69	<0.0001
Visceral adipose tissue mass for Mexican-Americans, kg	0.83 (0.08)	0.75 (0.08)	0.16	0.78 (0.09)	0.73 (0.09)	0.46	0.80 (0.06)	0.75 (0.06)	0.29	0.97	0.08
Visceral adipose tissue area for Koreans, cm^2^	78.8 (8.4)	82.1 (8.6)	0.54	71.8 (7.8)	56.2 (9.1)	0.04	81.4 (7.8)	73.3 (8.0)	0.08	0.34	0.08
Total mass, kg	86.14 (1.02)	86.68 (1.02)	0.006	85.80 (1.02)	85.94 (1.02)	0.53	85.33 (1.01)	85.25 (1.01)	0.68	0.84	0.11

All measures were adjusted for age, BMI, ethnicity, and interaction terms except for BMI itself.
