# Erratum to “Effects of Exercise Training on Fat Loss and Lean Mass Gain in Mexican-American and Korean Premenopausal Women”

**DOI:** 10.1155/2019/8308475

**Published:** 2019-05-27

**Authors:** Shenghui Wu, Kyung-Shin Park, Joseph B. McCormick

**Affiliations:** ^1^Department of Epidemiology & Biostatistics, University of Texas Health Science Center at San Antonio-Laredo Campus, Laredo, TX 78041, USA; ^2^Kinesiology, Texas A&M International University, 5201 University Blvd., Laredo, TX 78041, USA; ^3^Division of Epidemiology, School of Public Health, Brownsville Campus, University of Texas Health Science Center-Houston, Brownsville, TX 78520, USA

In the article titled “Effects of Exercise Training on Fat Loss and Lean Mass Gain in Mexican-American and Korean Premenopausal Women” [1], during the production stage, there was a typing error in the Abstract and Conclusions sections, as the sentence “Exercise training had a beneficial effect on reducing BMI, fat percentage, fat mass, and visceral adipose tissue area but had no effect on increasing lean mass for Mexican-American and Korean premenopausal obese women” should be corrected as follows:

“Exercise training had a beneficial effect on reducing BMI, fat percentage, fat mass, and visceral adipose tissue area but increasing lean mass for Mexican-American and Korean premenopausal obese women.”
